# Retraction: Physical activity motivation is inversely associated with anxiety: a cross-sectional serial mediation analysis involving smartphone addiction symptoms and sleep quality in medical undergraduates

**DOI:** 10.3389/fpsyg.2026.1985173

**Published:** 2026-09-08

**Authors:** Frontiers Editorial Office

The Journal retracts the 12 March 2026 article cited above.

Following publication, concerns were raised regarding the unauthorized use of the copyrighted material, the Chinese version of the Sleep Quality Questionnaire (SQQ-C), used in this study. Specifically, during the investigation, which was conducted in accordance with Frontiers' policies, the authors failed to provide appropriate authorization for use of the Chinese version of the Sleep Quality Questionnaire (SQQ-C). Unauthorized use of copyrighted material is a breach of Frontiers' guidelines and those of the Committee on Publication Ethics. As such, this article is being retracted.

This retraction was approved by the Chief Executive Editor of Frontiers. The authors have not responded to correspondence regarding this retraction.

Frontiers would like to thank Runtang Meng for contacting the journal regarding the published article.

